# Special Issue: “Traumatic Brain Injury/Chronic Traumatic Encephalopathy as Cause of Alzheimer’s Disease: Physics and Molecular Biology in the Genesis of Neurodegeneration?”

**DOI:** 10.3390/ijms27052266

**Published:** 2026-02-28

**Authors:** Dimitrios Kanakis

**Affiliations:** 1 Laboratory of Pathology, Department of Basic and Clinical Sciences, University of Nicosia Medical School, Nicosia 2408, Cyprus; kanakis.d@unic.ac.cy; Tel.: +357-22471902; 2 Centre of Neuroscience and Integrative Brain Research (CENIBRE), University of Nicosia Medical School, Nicosia 2408, Cyprus

In recent years, interest has grown in clarifying the relationship between brain trauma and subsequent neurodegeneration. Current research increasingly aims to define pathogenetic mechanisms that could explain how traumatic brain injury (TBI) and/or chronic traumatic encephalopathy (CTE) may influence later neurodegenerative processes, potentially culminating in a clinical diagnosis of Alzheimer’s disease. Importantly, TBI/CTE and Alzheimer’s disease share clinical features and histopathological changes that warrant careful consideration. This Special Issue was conceived to examine this possible link and to contribute to a clearer interpretation of any potential cause-and-effect relationship between TBI/CTE and Alzheimer’s disease. The articles included here advance this goal by addressing these entities from multiple perspectives and methodological approaches, thereby enriching our understanding of their intersections and distinctions.

Katramadou and colleagues provide evidence connecting the physics of head trauma, especially rotational acceleration and tissue deformation, to diffuse axonal injury (DAI) and white matter vulnerability. These primary insults trigger secondary cascades related to AD-relevant biology: blood–brain barrier (BBB) disruption, neuroinflammation, neurovascular and mitochondrial dysfunction, and protein aggregation, with dementia risk rising in a dose-responsive manner with injury severity and repetition and moderated by factors such as ApoE4. The review underscores stage specific therapeutic interventions, spanning stem cell-based methods for BBB and neural repair, use of neuroprotective agents, mitochondrial stabilization and immunomodulation [Contribution 1].

Blaschke and colleagues introduce a closed-head concussive brain injury (CBI) mouse model designed to evaluate the integrity of the blood–brain barrier (BBB) after single and repetitive CBI. The disruption of BBB, which becomes evident as a result of the administered dextran and IgG extravasation, correlates with functional deficits and increases with injury frequency. In this study, multimodal assessment (MRI, PET with [18F]DPA-714, histology) links “mild” injury phenotypes to measurable vascular compromise [Contribution 2].

Joseph’s focused review argues that physiology-sensitive imaging, including late-phase perfusion analysis by 3D arterial spin labeling (ASL) MRI and complementary modalities, may provide empirical evidence of subtle BBB dysfunction and white matter shear injury. Using physiologic testing measures could improve predictions about when it is safe for athletes with mild traumatic brain injury (mTBI) to return to sport. Better understanding of BBB repair could further enable new therapies and outcome tracking, and detecting BBB dysfunction may also support earlier intervention in preclinical neurodegenerative disease [Contribution 3].

Neuschmid and colleagues focus on calcium homeostasis as a shared vulnerability across TBI and AD. Their review highlights calcium-driven enzyme networks (kinases, phosphatases, proteases) that promote synaptic dysfunction, axonal degeneration, amyloid-β accumulation, and tau hyperphosphorylation, with calpain positioned as a key regulator. The editorial claims for more precise therapeutic strategies (including the combined inhibition of selected calcium-dependent enzymes or downstream targets) along with improved TBI phenotyping, long-term observational studies, and polygenic risk scoring to better define dementia risk and guide precision interventions [Contribution 4].

Mitroshina and Vedunova underline the importance of hypoxia as a contributor to neurodegeneration, while emphasizing that HIF-1α involvement can be complex and context-dependent. An imbalanced HIF-1-mediated response to hypoxia may contribute to neurodegeneration. In Alzheimer’s disease (AD) and Parkinson’s disease (PD), HIF-1 can be protective in some contexts yet proinflammatory and neurotoxic in others. Its effects likely depend on the severity and duration of hypoxia and related conditions. For this reason, therapeutic strategies should aim to modulate HIF-1 carefully: boosting HIF-1 in early disease or mild hypoxia to support neuronal survival and reduce inflammation, but potentially suppressing it in chronic hypoxia or advanced AD where harmful effects may dominate [Contribution 5].

Egoraeva and colleagues provide a concrete example of multi-process repair in a mouse model of mild traumatic brain injury (mTBI) using N-stearidonoylethanolamine (SDEA), an ω-3 ethanolamide. SDEA reduces astrocytic reactivity, restores Arc expression, improves CA1 dendritic spine density and morphology, and partially rescues dentate gyrus proliferative activity (Ki-67). These molecular and cellular changes coincide with improvements in anxiety-like behavior and working-memory performance. Overall, these findings suggest that ω-3 ethanolamides could be promising candidates for multi-target therapeutic approaches in mTBI [Contribution 6].

Pszczołowska and colleagues explicitly explore CTE in relation to AD, highlighting overlapping neuropathological features (notably tau- and amyloid-related themes) while emphasizing differences, including BBB-related damage patterns. The authors synthesize the currently available evidence on potential therapeutic options for chronic traumatic encephalopathy (CTE) and conclude that future progress will likely depend on two complementary priorities: improved prevention of traumatic brain injuries (TBIs) and the development of more effective approaches to limit neuroinflammation. An optimal therapeutic strategy would target the cellular and molecular drivers of secondary injury and cell death, including immune cells, astrocytes, and key inflammatory mediators such as cytokines and chemokines [Contribution 7].

Volloch and Rits-Volloch push toward theory revision. Their perspective traces the evolution of an “Amyloid Cascade Hypothesis 2.0” toward a model centered on sustained neuronal integrated stress response (ISR) biology. They present C99 and intraneuronal amyloid-related species as key drivers and propose that proteolytic A-beta production is suppressed in AD-affected neurons under ISR conditions. They argue this should reshape the interpretation of model systems and therapeutic strategy, including skepticism that extracellular A-beta-focused approaches will reverse symptomatic disease, and propose alternatives aimed at ISR modulation and targeted degradation pathways [Contribution 8].

